# Supplementary material for: Solid-state 1H spin polarimetry by 13CH3 nuclear magnetic resonance
Source: Magn Reson (Gott). 2021 Aug 20;2(2):643–52. doi: 10.5194/mr-2-643-2021 (PMC10539844; doi:10.5194/mr-2-643-2021)
Supplement: The supplement related to this article is available online at: https://doi.org/10.5194/mr-2-643-2021-supplement. [file mr-2-643-supplement.pdf]

Supplement of Magn. Reson., 2, 643–652, 2021
https://doi.org/10.5194/mr-2-643-2021-supplement
© Author(s) 2021. CC BY 4.0 License.

Open Access
MAGNETIC RESONANCE

*Supplement of*

# Solid-state $^{1}H$ spin polarimetry by $^{13}CH_3$ nuclear magnetic resonance

**Stuart J. Elliott et al.**

*Correspondence to:* Stuart J. Elliott (stuart.elliott@liverpool.ac.uk)

The copyright of individual parts of the supplement might differ from the article licence.

Contents

1 $^{13}C$ NMR Spectra 3

2 $^{13}C$ NMR Peak Normalized CoG Deviation vs. $^{1}H$ Polarization 4

## 1. $^{13}$C NMR Spectra

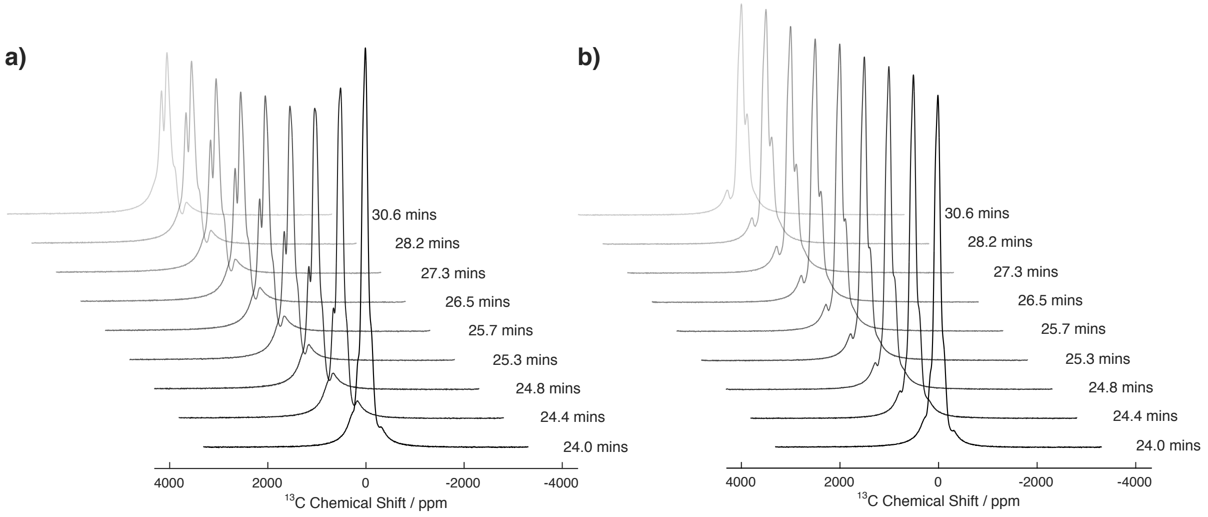


Figure S1: Relevant portions of the experimental $^{13}$C NMR spectra belonging to the $^{13}$C-labelled methyl group ($^{13}CH_3$) of [2-$^{13}$C]sodium acetate acquired at 7.05 T ($^{1}$H nuclear Larmor frequency = 300.13 MHz, $^{13}$C nuclear Larmor frequency = 75.47 MHz) and 1.2 K with a single transient (*rf*-pulse flip angle = 3.5°) as a function of $^{1}$H DNP time. (a) Positive microwave irradiation; and (b) Negative microwave irradiation. The labels indicate the $^{1}$H DNP time at which the spectra were recorded.

Figure S1 shows the relevant part of the experimental $^{13}$C NMR spectra acquired with a small flip angle *rf*-pulse ($\beta$ = 3.5°) as a function of $^{1}$H DNP time. The $^{13}$C NMR spectra in Figure S1 were acquired by using the *rf*-pulse sequence shown in Figure 1 of the main text. The timings coincide with those shown in Figure 2 of the main text.

## 2. $^{13}$C NMR Peak Normalized CoG Deviation vs. $^{1}$H Polarization

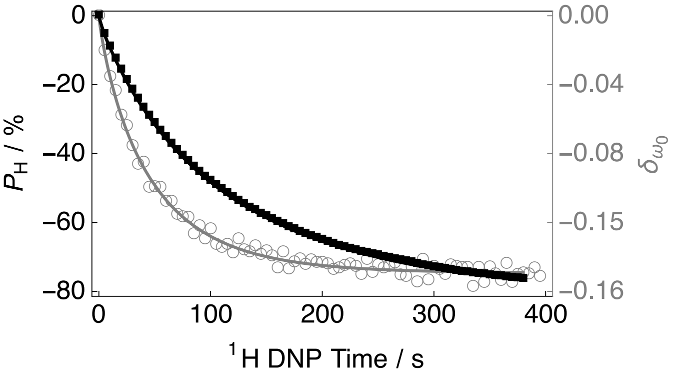


Figure S2: Experimental $^{1}$H polarization $P_H$ DNP build-up curve (black filled squares) and $^{13}$C NMR peak normalized CoG deviation $\delta_{\omega_0}$ (grey empty circles) as a function of $^{1}$H DNP time acquired at 7.05 T ($^{1}$H nuclear Larmor frequency = 300.13 MHz, $^{13}$C nuclear Larmor frequency = 75.47 MHz) and 1.2 K with a single transient per data point for the case of negative microwave irradiation. The timings coincide with those shown in Figure 2 of the main text. The black solid line indicates the best fit of the experimental data points for the $^{1}$H polarization $P_H$ DNP build-up curve, and has the corresponding fitting function: A(1-exp{-(t/$\tau_{DNP}^{-}$)$^{\beta}$}). Mean $^{1}$H DNP build-up time constant: $\langle\tau_{DNP}^{-}\rangle$ = 122.0 ± 0.4 s.

Figure S2 shows the DNP build-up curve for the $^{1}$H polarization $P_H$ as a function of $^{1}$H DNP time for negative microwave irradiation. Figure S2 also displays the $^{13}$C NMR peak normalized CoG deviation $\delta_{\omega_0}$ as a function of $^{1}$H DNP time.

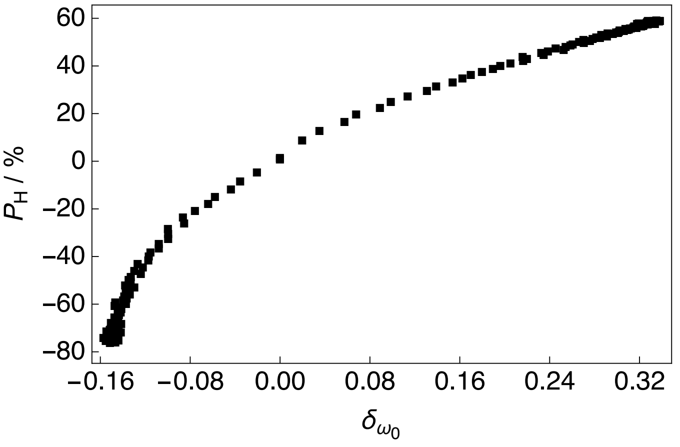


Figure S3: Experimental $^{1}$H polarizations $P_H$ as a function of the $^{13}$C NMR peak CoG normalized deviation $\delta_{\omega_0}$ acquired at 7.05 T ($^{1}$H nuclear Larmor frequency = 300.13 MHz, $^{13}$C nuclear Larmor frequency = 75.47 MHz) and 1.2 K with a single transient per data point. The absolute $^{1}$H polarizations $P_H$ were measured by comparison with a thermal equilibrium $^{1}$H NMR signal.

Figure S3 shows the $^{13}$C NMR peak CoG normalized deviation $\delta_{\omega_0}$ for sample **I** as a function of the $^{1}$H polarization $P_H$. An overall trend could be gleamed from this data set by fitting the experimental curve with an $n^{th}$ order spline function.
